# Diffused Aneurism of the Popliteal Space Healed by Wide Excision of Arteries and Veins

**Published:** 1918-12

**Authors:** 


***Diffused Aneurism of the Popliteal Space Healed by Wide Excision of Arteries and Veins.*** By MM. Moncany and Legendre. *Bulletin de la Société de Chirurgie de Paris,* May 7, 1918.

A soldier wounded in the right leg, the 11th of June, by grenade fragments, was operated the 15th of July, for an aneurism. A wide and long incision was made on the median line of the leg, from the end of popliteal space to the inferior third of the calf of the leg. A clot as big as the fist was discovered, and many other clots existed in the popliteal space. All the vessels and nerves were enclosed in thick fibrous tissues. 14 cm. of vessels were removed, of which 5 cm. were from the fibular and posterior tibial arteries.

It was found :

1) That the veins were not affected, but could not be separated from the arteries;

2) That the posterior tibial artery showed an aneurism of sacculated form, as big as a pigeon's egg. It seems that the bursting of this localized aneurism was the cause of the diffused one. The wound healed, but in January still presented edema. The authors insist on this operation; the ligature of the tibial-fibular vessels is considered as a dangerous one. But in this case, add the authors, anastomosis had had time to develop so as to assure the blood circulation.

